# The Future of Bladder Cancer Therapies: Integrating Hydrogel Scaffolds for Epigenetic Regulator Delivery

**DOI:** 10.7759/cureus.95675

**Published:** 2025-10-29

**Authors:** Kayode A John, Augustine O Odibo, Aliaa M Soliman, Modinat Abayomi, Favour M Awah, Vincent U Barrah

**Affiliations:** 1 Biomedical Sciences, University of Texas Health Science Center at San Antonio, San Antonio, USA; 2 Tissue Engineering, Villanova University, Villanova, USA; 3 Psychology, Austin Peay State University, Clarksville, USA; 4 Biology, Boston College, Boston, USA; 5 Biochemistry, City University of New York (CUNY) Graduate Center, New York, USA; 6 Public Health, Chicago State University, Chicago, USA

**Keywords:** chemoablation, gelma hydrogels, hydrogel drug delivery, mucoadhesive polymers, thermosensitive hydrogels

## Abstract

Urine rapidly dilutes and eliminates intravesical chemotherapies, making non-muscle-invasive bladder cancer (NMIBC) a clinical challenge. Reverse thermal hydrogels, mucoadhesive polymers, and tissue-engineered scaffolds have been developed to improve dwell time and target drug delivery. Recent Food and Drug Administration-approved hydrogel formulations have achieved high complete response rates in low-grade intermediate-risk NMIBC. At the same time, dysregulated epigenetic regulators such as lysine demethylase 6A and enhancer of zeste homolog 2, as well as methylation signatures detectable in urine, are revealing new therapeutic and diagnostic avenues. This narrative review synthesizes evidence on hydrogel-based delivery and scaffolding, arguing that combining sustained-release platforms with local epigenetic therapy and regenerative scaffolds represents the next frontier in NMIBC treatment, potentially reducing recurrence while improving bladder preservation.

## Introduction and background

Bladder cancer is the ninth most common cancer worldwide [1], and non-muscle-invasive disease (stages Ta, T1, and carcinoma in situ) accounts for about 70% of cases [2]. Standard management involves transurethral resection of bladder tumor (TURBT) followed by intravesical Bacillus Calmette-Guérin (BCG) or chemotherapies. Despite careful resection, over half of patients with low-grade intermediate-risk non-muscle invasive bladder cancer (NMIBC) recur within one year [3,4]. The challenge arises because the bladder is a dynamic reservoir lined with a mucin-rich glycosaminoglycan (GAG) layer that protects the underlying urothelium. Instilled drugs are quickly diluted by urine and voided; only a fraction permeates the GAG layer. Repeat surgeries subject patients to anesthesia and carry cumulative morbidity.

To overcome these pharmacokinetic challenges, three complementary strategies have emerged. First, hydrogel drug carriers exploit stimulus-responsive polymers to prolong bladder wall contact and sustain local drug concentrations. Second, epigenetic insights reveal dysregulated chromatin modifiers as both therapeutic targets and sources of urinary biomarkers for surveillance. Third, tissue-engineering scaffolds provide regenerative platforms that could lower the chance of recurrence after surgery by restoring the normal structure of the bladder. These approaches converge on a common goal: transforming brief, diluted drug exposure into sustained, localized therapy while promoting functional tissue repair.

Hydrogel carriers offer a means to improve retention and local drug concentration. Reverse-thermal gels (such as poloxamer-based RTGel®) remain fluid at room temperature but solidify at body temperature, forming a depot that adheres to the bladder wall. UroGen’s ZUSDURI (formerly UGN-102), containing mitomycin and a proprietary thermogel, recently became the first intravesical medication for adults with recurrent low-grade intermediate-risk NMIBC to receive Food and Drug Administration (FDA) approval. This approval was granted on June 12, 2025 [5]. The FDA Oncologic Drugs Advisory Committee (ODAC) voted 5-to-4 against the benefit/risk profile in May 2025, but the FDA still approved the drug in June 2025 [6]. In the Phase 3 ENVISION trial, 79.6% of patients achieved a complete response at three months, and 79% of those respondents remained event-free one year later [7]. A postapproval analysis reported that out of 223 evaluable participants, 78% had a complete response and 79% of respondents stayed cancer-free for at least a year; common adverse events included dysuria, urinary tract infection, and hematuria [7]. A long-term follow-up of the OPTIMA II trial estimated a median response duration of 24.2 months [8].

Mucoadhesive polymers, particularly chitosan, also extend dwell time by binding to the negatively charged mucin layer. Chitosan nanoparticles accumulate in the GAG layer, forming a microconcentration gradient that enhances diffusion into the bladder wall [9]. Paclitaxel-loaded chitosan nanosuspensions show high drug loading, positive surface charge, strong mucin adhesion, and sustained release; in mice, the nanosuspension reduced tumor volume and increased necrotic areas compared with paclitaxel/chitosan blends [9]. Other strategies, such as floating hydrogels (e.g., adding bicarbonate to generate microbubbles), aim to reduce urethral obstruction while maintaining contact with the urothelium.

Epigenetic regulation is also intimately linked to bladder carcinogenesis. Loss of the X-linked histone demethylase lysine demethylase 6A (KDM6A), an H3K27 demethylase, occurs in a large fraction of urothelial carcinomas. Recent work showed that KDM6A or forkhead box A1 (FOXA1) deficiency triggers an activating transcription factor 3 (ATF3)-driven transcriptional program that promotes cell proliferation and inflammatory signaling, while KDM6A/FOXA1 normally restrains this circuit. Methylation signatures are being harnessed for post-TURBT surveillance; in a prospective, blinded, multicenter North American study of 449 NMIBC patients undergoing cystoscopic surveillance, the Bladder EpiCheck urine test, which analyzes 15 methylation loci, demonstrated an overall sensitivity of 68%, high-grade sensitivity of 89%, and specificity of 88% [10]. These findings suggest that localized epigenetic modulators delivered via hydrogels could complement chemotherapeutic depots, while methylation markers can guide patient selection and monitoring. Whether baseline or longitudinal methylation profiles correlate with response to hydrogel-based therapy remains an important area for future investigation.

**Figure 1:**
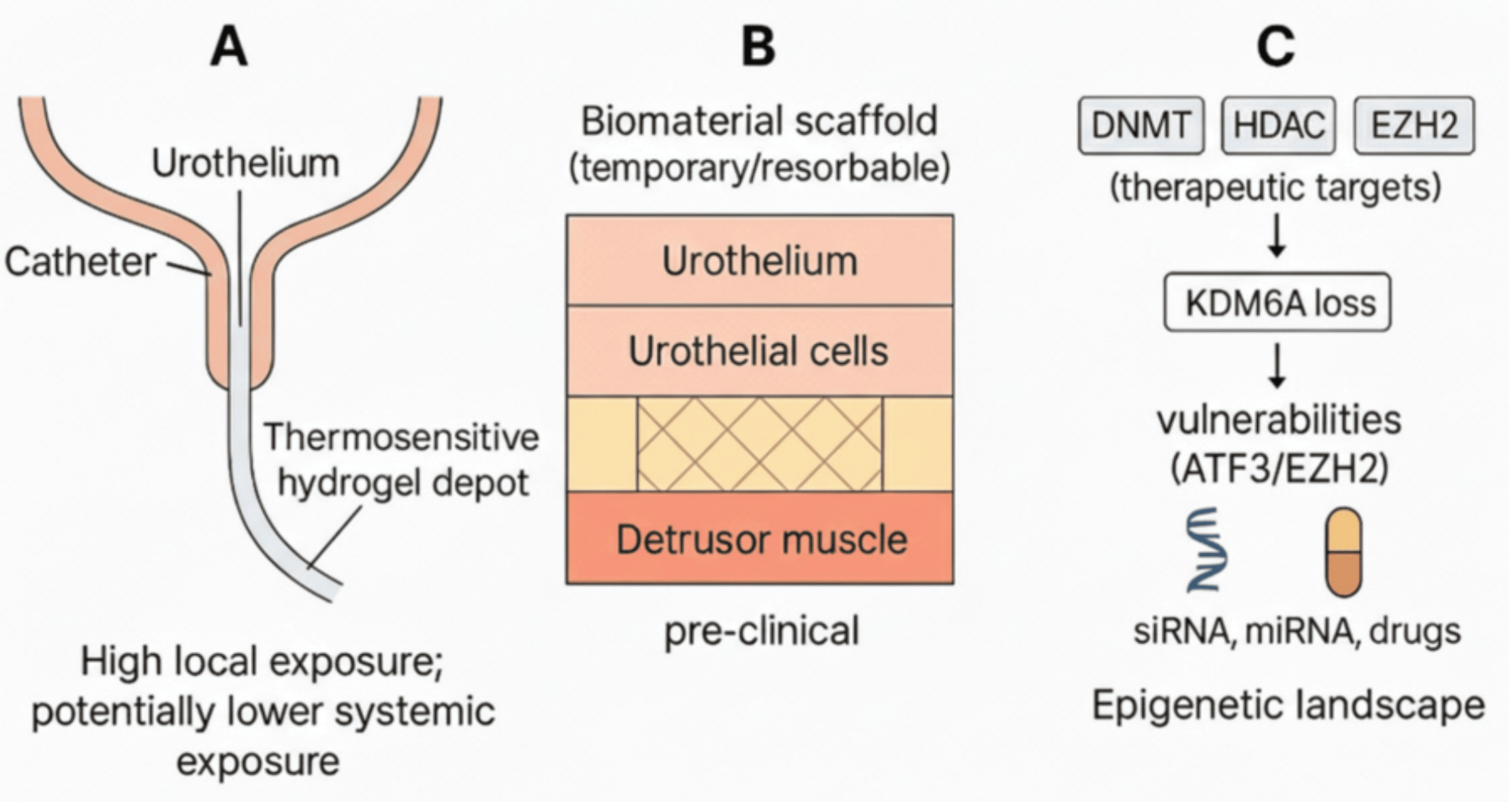
Hydrogel-epigenetic convergence in bladder cancer (A) Intravesical catheter delivers a thermosensitive hydrogel that gels on the urothelium, providing high local exposure with potentially lower systemic exposure. (B) Temporary/resorbable biomaterial scaffold supporting urothelial cells and detrusor architecture (preclinical). (C) Epigenetic landscape: therapeutic targets (DNMT, HDAC, EZH2) and vulnerabilities from KDM6A loss (ATF3/EZH2); local vectors (siRNA, miRNA, drugs). Structures represent simplified schematic representations for illustrative purposes and are not to scale DNMT: DNA methyltransferase; HDAC: histone deacetylase; EZH2: enhancer of zeste homolog 2; KDM6A: lysine demethylase 6A; ATF3: activating transcription factor 3 Image credit: This is an original image created by the author Vincent U. Barrah using DALL-E for initial conceptualization, with refinement in BioRender

The tissue-engineering triad (cells, scaffolds, and biochemical cues) underpins regenerative strategies. Biocompatible hydrogels such as gelatin methacryloyl (GelMA) can be molded into scaffolds that mimic the extracellular matrix. In a murine model of cyclophosphamide-induced cystitis, a GelMA granular hydrogel loaded with periostin promoted urothelial regeneration by upregulating Wingless/Int (Wnt) signaling and polarizing macrophages toward a reparative type-2 macrophage (M2) phenotype [11]. Decellularized extracellular matrix (dECM), silk-fibroin, and electroactive polymers are also being investigated for bladder reconstruction. Figure 1 provides a conceptual overview of the therapeutic and engineering interfaces. This review integrates these themes to examine how hydrogel drug carriers, epigenetic insights, and tissue engineering scaffolds can converge to improve NMIBC outcomes.

## Review

Methods

A narrative literature review was conducted. Searches were performed in MEDLINE/PubMed, Embase, Scopus, and the Web of Science for articles published from 2015 to August 2025. Keywords included “bladder cancer”, “urothelial carcinoma”, “hydrogel”, “thermogel”, “mucoadhesive”, “intravesical”, “tissue engineering”, “scaffold”, “GelMA”, “decellularized matrix”, “epigenetic”, “DNA methylation”, “histone modification”, “EZH2”, “KDM6A”, “HDAC”, and “DNMT”, combined with Boolean operators. Original research articles, clinical trials, or prospective/retrospective cohort studies; studies reporting on hydrogel formulations for intravesical therapy, tissue engineering scaffolds for bladder repair, or epigenetic mechanisms/biomarkers in urothelial carcinoma; and preclinical studies with relevant mechanistic insights or translational implications were included. Conference abstracts and letters without original data; non-peer-reviewed content; studies not available in English; and articles focused solely on muscle-invasive or metastatic bladder cancer without NMIBC relevance were excluded. Reference lists of relevant articles were manually screened for additional sources. Data extraction focused on hydrogel formulations for intravesical therapy, scaffold materials for bladder repair, epigenetic mechanisms and biomarkers, and clinical trial outcomes. No meta-analysis was performed. Searches were executed on August 12, 2025.

Results

Evidence from both preclinical and clinical studies demonstrates the progression of hydrogel and scaffold technologies from concept to clinical use. The following sections examine intravesical hydrogel carriers, clinical trial results, regenerative scaffolds, and the epigenetic landscape, highlighting safety, efficacy, and durability.

Hydrogel Carriers for Intravesical Therapy

Reverse-thermal hydrogels such as poloxamer RTGel® have enabled a paradigm shift from immediate drug washout to sustained chemoablation. ZUSDURI delivers mitomycin within a thermogel that solidifies at body temperature. Kaplan-Meier analysis estimated that responders had an 80.6% probability of remaining event-free 18 months later [8]. Adverse events were mostly grade 1-2 dysuria (22.5%), hematuria (8.3%), urinary tract infection (7.1%), and fatigue (5.4%); serious events included urethral stenosis and urinary retention in 0.4% each [8]. UGN-101 (Jelmyto®) used a similar gel for upper tract urothelial carcinoma and showed a 59% complete response rate after six weekly instillations in a phase 3 trial [12].

Mucoadhesive hydrogels utilize natural polymers (chitosan, hyaluronic acid) that bond to mucin. Chitosan nanoparticles create a microconcentration gradient, enhancing diffusion and increasing survival compared with commercial BCG formulations [9]. Paclitaxel-chitosan nanosuspensions improved mucin adhesion and maintained high drug loading; in mice, the nanosuspension produced greater tumor necrosis than paclitaxel/chitosan blends [9]. Magnetic chitosan/β-glycerophosphate hydrogels (containing Fe_3O_4) have been used to deliver BCG; the magnetic field holds the gel against the bladder wall and improves immunotherapy durability. However, chitosan formulations require careful optimization, as excessive concentrations may cause urothelial irritation. The mechanisms of potential toxicity, optimal concentration ranges for intravesical use, and long-term biocompatibility remain areas requiring further investigation before clinical translation. Clinical dose-finding and safety studies will be essential to establish therapeutic windows for chitosan-based intravesical platforms.

Floating thermogels are another innovation to address bladder dynamics. By incorporating gas-generating agents such as sodium bicarbonate or perfluoropropane, these gels form microbubbles that make the depot buoyant. The resulting formulation maintains contact with the urothelium for more than two hours and reduces the risk of urethral obstruction [13]. Although still preclinical, floating gels illustrate how physical properties can be tuned to meet anatomical constraints.

Clinical Translation of Hydrogel-Based Intravesical Chemoablation (LG-IR NMIBC)

Chemoablation (UGN-102/ZUSDURI): In ENVISION, 240 patients received six weekly instillations of mitomycin (1.33 mg/mL); 228 completed all doses, and 79.6% achieved a complete response at three months [14]. Among responders, the 18-month probability of remaining in response was ~80.6% [7], and 24-month durability was ~72.2% in program updates [15]. On June 12, 2025, the FDA approved mitomycin intravesical solution (ZUSDURI) for adults with recurrent low-grade, intermediate-risk (LG-IR)-NMIBC [7,15]. Table 1 summarizes key efficacy, durability, and safety outcomes across these pivotal hydrogel-based intravesical therapy trials.

**Table 1:** Clinical trial outcomes for hydrogel-based intravesical therapy Summary of pivotal phase 3 trials evaluating mitomycin-containing reverse thermal hydrogels for NMIBC (ENVISION, ATLAS) and upper tract urothelial carcinoma (OLYMPUS) NR: not reported in text; DoR: duration of response; DFS: disease-free survival; UTI: urinary tract infection; TURBT: transurethral resection of bladder tumor; CTCAE: Common Terminology Criteria for Adverse Events

Study	Agent	Design	n	Complete response	Duration/survival	Key adverse events
ENVISION [7,14]	UGN-102 (mitomycin gel)	Single-arm, phase 3	228	79.6% (3 months)	Median DoR: 24.2 months; 18-month event-free 80.6%	Urethral stenosis (0.4%), retention (0.4%); Grade 1-2: dysuria (22.5%), UTI (7.1%), hematuria (8.3%)
ATLAS [16]	UGN-102 ± TURBT vs. TURBT alone	Randomized, phase 3	NR	65% vs. 64% (3 months)	15-month DFS: 72% vs. 50%	NR
OLYMPUS [17]	UGN-101 (upper tract)	Single-arm, phase 3	NR	59% (primary evaluation)	NR	Ureteral stenosis, hydronephrosis

Randomized evidence (ATLAS): In the phase 3 ATLAS trial, three-month CR was similar (65% vs. 64%), while 15-month disease-free survival favored UGN-102 ± TURBT (72% vs. 50%) [16]. Regulatory briefing materials highlighted interpretability concerns and the role of randomized evidence in this setting [18].

Regulatory context: The FDA's ODAC voted 5-to-4 against the overall benefit-to-risk before approval; postmarketing evidence expectations were noted [19].

**Figure 2:**
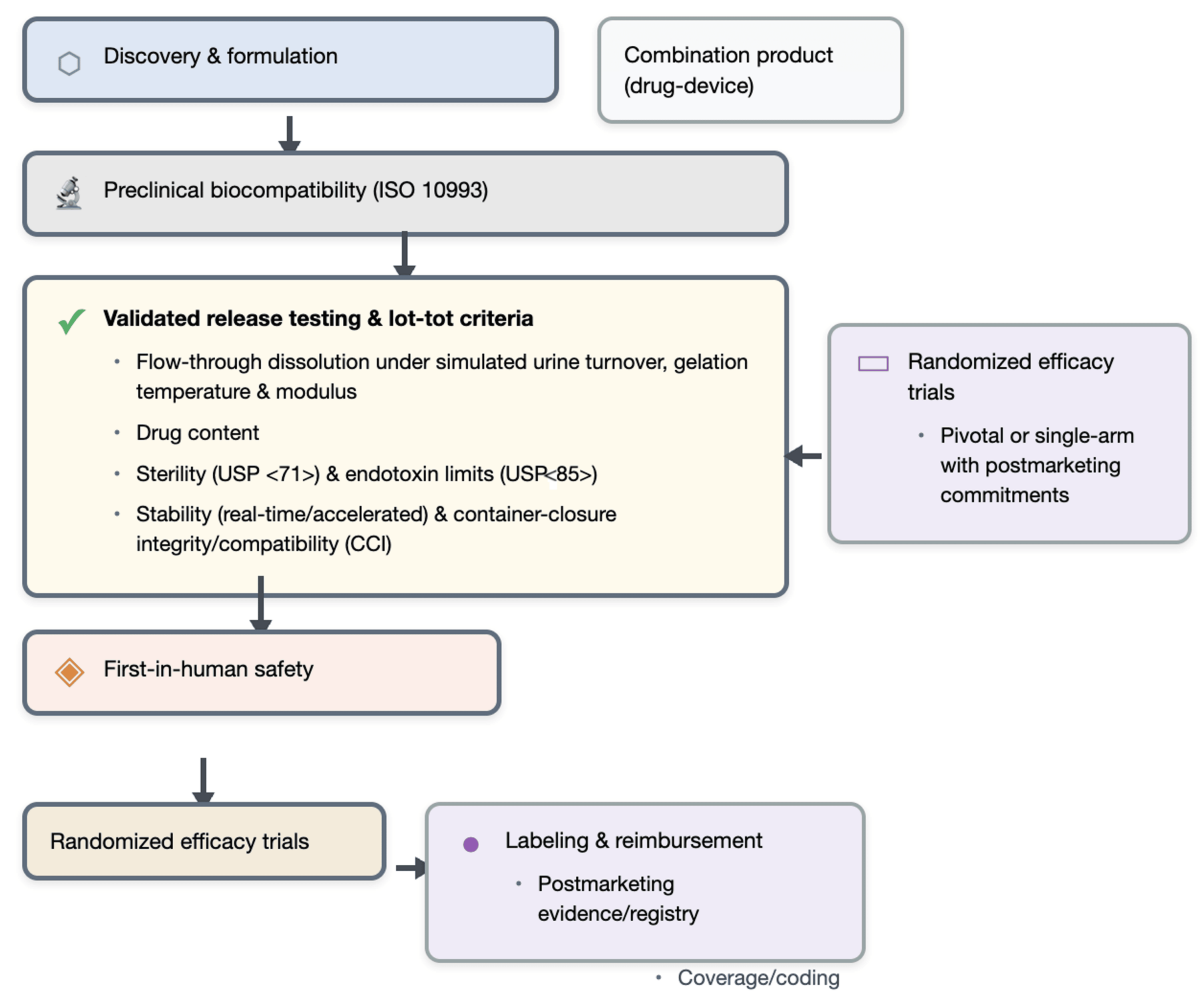
Translation pathway for hydrogel combination products The translation pathway begins with discovery and formulation, including formal designation as a combination product, then proceeds to preclinical biocompatibility testing in accordance with International Organization for Standardization 10993. Next, manufacturing readiness is proven through validated release and quality gates under urine-turnover conditions: flow-through dissolution, gelation temperature and modulus, drug-content assay, sterility per USP <71>, endotoxins per USP <85>, stability studies, and container-closure integrity. After these gates are met, development advances to first-in-human safety evaluation and then to pivotal efficacy testing, typically randomized trials or a single-arm study with defined postmarketing commitments. Successful results enable labeling and reimbursement, which are sustained by postmarketing evidence or registries alongside coverage and coding activities USP: United States Pharmacopeia Image credit: This is an original image created by the author Vincent U. Barrah using DALL-E for initial conceptualization, with refinement in BioRender

Upper tract precedent (UGN-101/Jelmyto): The phase 3 OLYMPUS trial in low-grade UTUC reported a 59% CR at primary evaluation after six weekly instillations [17]. The stage-gate path from discovery through International Organization for Standardization (ISO) 10993 biocompatibility and validated release testing to labeling and reimbursement is summarized in Figure 2.

Hydrogel Scaffolds for Bladder Repair

Beyond drug delivery, hydrogels serve as scaffolds for tissue regeneration. GelMA hydrogels loaded with periostin promoted urothelial regeneration and M2 macrophage polarization in a cyclophosphamide-induced cystitis model [11]. dECM derived from porcine bladder retains collagen and elastin and can be seeded with urothelial and smooth-muscle cells [20,21]. Silk-fibroin hydrogels offer mechanical strength and elasticity [22]; combining them with growth factors or dECM components can improve cell adhesion and differentiation [23]. Electroactive polymers can deliver electrical cues to align smooth muscle and are being explored in 3D-printed bladder constructs [24]. Reverse-thermal hydrogels have also been applied to the upper urinary tract: in a phase 3 trial, UGN-101 (Jelmyto®) delivered mitomycin to the pyelocaliceal system and achieved nearly 50% complete remissions after six weekly instillations [13]. Although promising, these scaffolds and combination products remain largely preclinical or early clinical, with challenges including vascularization, innervation, and long-term biomechanical integration. Notably, the final analysis of the OLYMPUS trial reported a 59% complete response rate for UGN-101 [25].

Epigenetic Landscape

Epigenetic dysregulation is pervasive in urothelial carcinogenesis; recurrent alterations include DNA methyltransferase (DNMT) and histone deacetylase (HDAC) activity, as well as polycomb repressive complex 2 (PRC2)/enhancer of zeste homolog 2 (EZH2) signaling, alongside frequent loss of KDM6A and FOXA1 in NMIBC [26]. KDM6A loss triggers an ATF3-dependent switch that disrupts urothelial differentiation and promotes proliferation and inflammatory signaling; ATF3 knockdown reverses this phenotype [27]. Overexpression of the methyltransferase EZH2 and mutations in ARID1A or KDM6A may sensitize tumors to EZH2 inhibitors. DNA methylation panels, such as Bladder EpiCheck, achieve high specificity (88%) and high-grade sensitivity (89%) for detecting recurrence [10]. Although DNMT and HDAC inhibitors are approved for hematologic malignancies, their systemic toxicity limits intravesical use. Localized delivery via hydrogels could allow lower doses and reduce systemic exposure. Preclinical studies are exploring miRNA- or small interfering RNA (siRNA)-loaded hydrogels to suppress oncogenic drivers.

Discussion

The randomized signal suggests chemoablation may extend disease-free intervals vs. TURBT alone in selected low-grade, intermediate-risk NMIBC [28]. However, early termination and nonidentical disease-free survival definitions limit inference [16].

Engineering Criteria for Bladder-Compatible Hydrogels

Beyond these general requirements, polymer architecture and cross-linking chemistry strongly influence performance. Poloxamer triblock copolymers can be blended with poly(lactide-co-glycolide)-poly(ethylene glycol)-poly(lactide-co-glycolide) triblocks to fine-tune gelation temperature, mechanical strength, and degradation rate [29,30]. pH-responsive hydrogels are based on poly(acrylic acid) or alginate gel in the acidic environment of urine and liquefy at neutral pH. In contrast, ion-sensitive hydrogels cross-link in the presence of divalent cations such as calcium. Catechol functional groups inspired by mussel foot proteins provide strong mucoadhesion via oxidative cross-linking with mucin and can be grafted onto chitosan or polyethylene glycol. Viscosity must be optimized: high polymer concentrations improve adhesion and prolong dwell time but reduce syringeability. Shear-thinning formulations allow injection through a catheter and recover high viscosity once shear stress ceases. Controlled degradation can be achieved by adjusting cross-link density or incorporating hydrolysable ester bonds; ideally, the gel persists long enough to deliver its payload but dissolves before significant voiding obstruction occurs. Safety considerations include minimizing residual monomers, endotoxins, and solvent residues and ensuring degradation products are excreted without toxicity. Physical innovations such as microbubble doping or hollow microfibers allow the tuning of density and buoyancy and may permit alternating contact with different bladder regions during filling and emptying cycles.

Mucoadhesion and stimulus responsiveness are paramount. The gel should remain liquid during instillation, gel at body temperature, adhere to mucin, and then degrade or dissolve without obstructing urine flow. Poloxamer RTGel® achieves a reversible sol-gel transition around 25°C-37°C [31]. Chitosan provides positive charges that bind to negatively charged mucin, but high concentrations may irritate the urothelium. Floating hydrogels mitigate outflow obstruction by generating microbubbles using bicarbonate or perfluoropropane; buoyancy persists for over two hours while drugs diffuse slowly. Release kinetics must balance sustained exposure with minimal burst release, and gels should be syringable through a catheter. Biocompatibility testing (ISO 10993) should evaluate cytotoxicity, irritation, and degradation products.

From Epigenome to Scaffold Design

Scaffold material and architecture intimately interact with chromatin dynamics through mechanotransduction [32]. The extracellular matrix transduces stiffness and topographical cues to the nucleus via integrins and the cytoskeleton, modulating YAP/TAZ signaling [33] and histone acetylation [34]. For example, softer matrices tend to maintain epithelial differentiation, whereas stiffer matrices promote smooth-muscle lineage commitment. dECM derived from porcine bladder retains collagen [35], laminin, and growth factors such as fibroblast growth factor and heparan sulfate, providing biochemical cues that preserve urothelial identity [36]. Blending dECM with synthetic polymers like polycaprolactone or poly(lactic acid) improves mechanical strength and processability [37]. Three-dimensional bioprinting techniques enable deposition of cell-laden constructs with spatial control, generating multilayered structures that recapitulate the laminar organization of the bladder wall [38]. The GelMA-periostin scaffold study cited earlier [11] exemplifies this scaffold-epigenetics interface: the biomaterial activated Wnt signaling and shifted macrophage polarization, demonstrating how matrix composition influences cellular phenotype through chromatin-modulated pathways. Similarly, studies employing decellularized matrices in bladder reconstruction models have shown preservation of urothelial differentiation markers, consistent with extracellular matrix-mediated epigenetic stability [20,21]. While direct evidence linking specific scaffold parameters to histone modification patterns in bladder models remains limited, the mechanotransduction-chromatin axis is well-established in other tissue systems [32-34], providing a conceptual framework for future bladder-specific investigations. Microgrooved and electroactive scaffolds can align smooth-muscle cells and deliver electrical stimuli, respectively; these physical cues feed back on chromatin state and gene expression [39,40]. Incorporating antifibrotic agents (e.g., bone morphogenetic protein inhibitors) or anti-inflammatory molecules into scaffolds may counteract the profibrotic epigenetic switch triggered by KDM6A loss. Because KDM6A resides on the X chromosome and its paralogue UTY on the Y chromosome has weak demethylase activity, male patients may be more susceptible to histone hypermethylation [41]; personalized scaffolds could contain epigenetic modulators or microRNAs tailored to the patient's sex and tumor epigenotype.

Epigenetic programs guide urothelial differentiation and may influence scaffold choice. Loss of KDM6A fosters a proliferative, inflammatory state, suggesting that scaffolds with anti-inflammatory cues could be beneficial [42]. Decellularized matrices preserve native basement membrane proteins and growth factors that maintain lineage fidelity. GelMA scaffolds loaded with periostin activate Wnt signaling and promote regeneration [11]. Sex-linked biology, e.g., KDM6A located on the X chromosome, suggests that female and male patients may respond differently to epigenetic modulation and scaffold cues. Profiling residual urothelium might inform scaffold selection and adjuncts: methylation signatures could identify aggressive lesions requiring drug-laden scaffolds; ATF3-activated tumors may benefit from local ATF3 suppression.

Localized Epigenetic Modulation via Hydrogels

DNMT inhibitors demethylate promoter regions of tumor-suppressor genes and can reactivate expression of p16, RASSF1A, or E-cadherin [43]. Low-dose decitabine has been used to prime solid tumors for immunotherapy by increasing antigenicity; intravesical delivery may replicate these effects while avoiding cytopenias associated with systemic exposure [44]. HDAC inhibitors acetylate histones and nonhistone proteins; valproic acid has been shown to restore cell-cycle inhibitors and inhibit proliferation in bladder-cancer models, and it has been explored clinically in non-muscle-invasive disease [45]. Local delivery could circumvent central nervous system side effects that limit systemic dosing [46]. EZH2 inhibitors block the methyltransferase activity of PRC2, reducing H3K27 trimethylation and derepressing genes involved in antigen presentation and differentiation; combination with checkpoint inhibition is under evaluation (e.g., tazemetostat plus pembrolizumab in advanced urothelial carcinoma) [47,48]. Beyond small-molecule inhibitors, microRNAs, such as miR-200c, regulate epithelial-mesenchymal transition, and miR-34a modulates PD-L1 expression [49,50]. Their local delivery via hydrogels could suppress invasion and enhance immune recognition [51]. Clustered, regularly interspaced short palindromic repeats (CRISPR)/catalytically inactive Cas9 (dCas9)-based epigenome editors, which tether dCas9 to demethylase or acetyltransferase domains, enable targeted editing of specific loci; hydrogels could protect these ribonucleoprotein complexes and provide sustained release [52,53]. Challenges include the need for efficient penetration through the GAG layer, minimizing off-target edits, and ensuring that epigenetic changes are durable yet reversible if adverse effects occur.

Delivering epigenetic drugs intravesically allows high local concentration with reduced systemic exposure [46]. Candidate payloads include DNMT inhibitors (decitabine, azacitidine), HDAC inhibitors (valproic acid, vorinostat), EZH2 inhibitors (tazemetostat), and siRNAs targeting oncogenic long noncoding RNAs [13]. For example, tazemetostat combined with pembrolizumab has shown activity in early clinical evaluation of advanced urothelial carcinoma [48]. Hydrogels can encapsulate these agents and release them slowly; nucleic-acid cargos can be protected by carriers such as chitosan or dendrimer complexes [51,54]. A tumor-selective mucoadhesive hydrogel employing a two-step pretargeting strategy with R11-biotin and streptavidin-modified nanoparticles achieved selective gelation on tumor surfaces and enhanced gemcitabine efficacy while minimizing off-target toxicity [55]. Safety assays should include genomic profiling for off-target methylation or histone modification changes, as well as assessment of immune activation [47].

Immunoepigenetic Synergy With Hydrogel Depots

At the mechanistic level, BCG triggers Toll-like receptor signaling and induces a cascade of cytokines, including IL-6, IL-12, and interferon-γ, that recruit innate and adaptive immune cells to the bladder [56]. Epigenetic drugs can upregulate major histocompatibility complex class I and II molecules and costimulatory ligands (CD80, CD86), thereby increasing the ability of tumor cells to present antigens and be recognized by cytotoxic T lymphocytes. DNMT or HDAC inhibition may also derepress endogenous retroelements, leading to a “viral mimicry” response that boosts type I interferon signaling. N-803, an interleukin-15 superagonist, enhances the proliferation and persistence of natural killer and CD8 T cells; early trials in BCG-unresponsive disease show encouraging complete response rates [57]. Combining N-803 with local epigenetic modulators could amplify antitumor immunity while limiting systemic toxicity [58]. Timing is critical: administering epigenetic priming days before BCG or checkpoint therapy allows upregulation of antigen-presentation machinery, whereas simultaneous delivery may lead to excessive inflammation or immunosuppression. Intravesical hydrogels can be engineered to release drugs sequentially, for example, by layering different polymers or encapsulating agents in microparticles with staggered degradation rates. In addition to adaptive immunity, epigenetic modulators can repolarize tumor-associated macrophages from an immunosuppressive M2 phenotype to a proinflammatory M1 phenotype and enhance dendritic cell maturation, creating a microenvironment conducive to BCG or checkpoint response.

BCG immunotherapy remains a cornerstone of NMIBC management; however, more than half of responders relapse. Epigenetic priming may enhance immunogenicity: DNMT or HDAC inhibitors can increase tumor antigen presentation and upregulate immune costimulators. Local delivery of epigenetic agents alongside BCG or interleukin-15 superagonists (e.g., N-803) could potentiate immune responses while limiting systemic toxicity. Murine studies show that chitosan-based hydrogels loaded with BCG prolong dwell time and improve survival [9]. Combining hydrogel depots containing miRNAs that downregulate PD-L1 or EZH2 inhibitors could synergize with checkpoint blockade. Scheduling may matter: epigenetic priming before BCG or checkpoint therapy could maximize antigenicity, whereas codelivery might risk excessive inflammation.

Translation Pathway: Manufacturing, Quality, and Regulation

Manufacturing practices for hydrogel combination products must follow current good manufacturing practices. Validated in vitro release (e.g., flow-through dissolution under simulated urine turnover) [59,60], predefined lot-to-lot criteria for gelation temperature/modulus/drug content [61], and sterility/endotoxin limits are essential for batch release and labeling consistency. Polymer synthesis requires controlled molecular weight distribution, polydispersity, and residual monomers; high-pressure liquid chromatography and gel permeation chromatography are used to verify purity and size. The drug loading process should ensure uniform distribution, stability during storage, and minimal leakage. Residual solvents and catalysts must be removed to levels below acceptable limits, and they must meet endotoxin limits (USP <85>) and pass sterility testing (USP <71>) [5,62]. For combination products containing cells or biologics, viral safety testing and screening for adventitious agents are required. In many jurisdictions, the regulatory pathway is determined by the product's primary mode of action [5]. A hydrogel carrying a chemotherapeutic is regulated primarily as a drug, whereas a scaffold seeded with autologous cells may be regulated as a biologic. Chemistry, manufacturing, and control submissions should include detailed descriptions of raw materials, synthesis steps, analytical methods, acceptance criteria, and stability data. Preclinical safety testing beyond ISO 10993 may include genotoxicity assays, repeated-dose toxicity, and the evaluation of potential leachables from polymers [63]. Large animal studies should assess urine flow, renal function, and tissue integration. Clinical trials should measure objective responses (complete and durable response, progression to muscle-invasive disease), functional outcomes (cystometry, urodynamics), health-related quality of life, and healthcare utilization. Health economic analyses should model costs and benefits, accounting for reduced surgery needs, fewer hospital visits, and improved productivity. Postmarketing surveillance through registries and electronic health records can capture long-term safety, adherence, and real-world effectiveness, thereby informing updates to guidelines and reimbursement policies.

Drug-device combination products such as ZUSDURI require compliance with both pharmaceutical and medical device regulations [64]. Manufacturing must ensure batch-to-batch consistency in polymer molecular weight, drug loading, and rheological properties. A sterile, low-endotoxin product is essential to minimize infection risk during intravesical administration [65,66]. ISO 10993 biocompatibility testing should address cytotoxicity, sensitization, irritation, systemic toxicity, and degradation [67]. Release assays must demonstrate reproducible kinetics under simulated bladder conditions. Clinical endpoints should include complete response, duration of response, recurrence-free survival, bladder function (cystometry), quality of life, and cost-effectiveness. Real-world evidence from registries like uTRACT will help refine coverage decisions [68]. Payer acceptance will depend on demonstrating that gel-based chemoablation reduces repeat surgeries and overall healthcare costs relative to TURBT [69].

Implications for Urologic Oncology, and Device Research and Development Leadership

For urologic oncologists and device development leaders, hydrogel therapies challenge traditional surgical paradigms. Clinic workflows must adapt to weekly instillations requiring catheterization and patient monitoring for dysuria or retention. Patient selection should focus on low-grade intermediate-risk NMIBC; high-grade lesions still warrant BCG or radical cystectomy. Coordination between urology, pharmacy, and device manufacturing is needed to ensure the timely preparation and administration of gel formulations. Cost considerations include the price of proprietary gels, procedural costs, and savings from avoiding repeat surgeries. While hydrogel-based chemoablation involves higher up-front drug acquisition costs compared to conventional intravesical instillations, preliminary health economic considerations suggest potential cost offsets through reduced recurrence rates and fewer repeat TURBT procedures. However, formal cost-effectiveness analyses incorporating quality-adjusted life years, bladder preservation rates, long-term surveillance costs, and real-world resource utilization are needed to inform payer coverage decisions and demonstrate value across diverse healthcare systems. Biomarker-guided surveillance, using urine methylation tests like Bladder EpiCheck [10], could reduce cystoscopy frequency and detect recurrence earlier. Leadership should also plan for training on new delivery devices and for collecting real-world data to support reimbursement and refine indications. For quick selection guidance, key hydrogel options and evidence tiers are summarized in Table 2.

**Table 2:** Emerging hydrogel-based synthesis for intravesical drug delivery: cargo, trigger, and designs FDA: Food and Drug Administration; UTUC: upper tract urothelial carcinoma; BCG: Bacillus Calmette-Guérin; GelMA: gelatin methacryloyl; dECM: decellularized extracellular matrix; Wnt: Wingless/Int

Material	Trigger/adhesion	Typical cargo	Evidence tier	Pros (keywords/phrases)	Cons (keywords/phrases)	Exemplar study/trial
Poloxamer RTGel (ZUSDURI/UGN-102)	Thermosensitive: liquid at low temperature, gels at 37°C	Mitomycin	Phase 3 clinical trial	Prolonged dwell, high complete response, first FDA approval	Six weekly instillations, dysuria, retention	ZUSDURI (UGN-102) ENVISION trial [7,8]
Poloxamer RTGel (Jelmyto/UGN-101)	Thermosensitive: liquid at low temperature, gels at 37°C	Mitomycin	Phase 3 UTUC trial	59% complete response; six weekly instillations	Risk of ureteric stenosis; ureteral obstruction	OLYMPUS final analysis [25]
Chitosan/hyaluronic-acid mucoadhesive hydrogels	Mucoadhesive via cationic charge binding mucin	Paclitaxel, BCG	Preclinical	High retention; sustained release; improved tumor necrosis	Potential urothelial irritation; limited clinical data	Paclitaxel-chitosan nanosuspension [9]
Floating thermogel	Thermosensitive poloxamer plus microbubbles for buoyancy	Mitomycin or other drugs	Preclinical	Reduces obstruction; maintains contact >2 hours [13]	Unproven in humans; complex formulation	Floating thermosensitive hydrogel concept [13]
GelMA/dECM scaffolds	Cross-linked hydrogel scaffold providing structural support	Periostin, cells	Preclinical animal models	Biomimetic; promotes regeneration; Wnt/M2 polarization	Early stage; variable degradation; surgical implantation	Periostin GelMA hydrogel study [11]
Tumor-selective mucoadhesive hydrogel	Two-step R11-biotin pretargeting and streptavidin-modified nanoparticles	Gemcitabine	Proof-of-concept [55]	Selective gelation; enhanced efficacy; reduced off-target toxicity	Requires ligand pretargeting; still preclinical	Tumor-selective gemcitabine hydrogel [55]

Because hydrogel instillations are performed in the clinic rather than the operating theater, scheduling and staffing must accommodate multiple catheterizations per patient over several weeks. Thorough patient counseling and adherence support are essential; remote monitoring via telehealth or smartphone applications can track symptoms and prompt return visits. Practices must develop infrastructure for compounding, cold-chain storage, and just-in-time mixing of drugs and polymers to preserve sterility and rheological properties. Nurses and technicians require training to recognize and manage adverse events, such as urinary retention, dysuria, and hematuria. Outcomes appear durable with manageable toxicity in trials, but comparative effectiveness, cost, and workflow impact will determine adoption at scale [5,14]. Selection remains anchored in low-grade, intermediate-risk cohorts, with high-grade disease managed per guideline-concordant pathways [70]. Clinicians should counsel patients about both efficacy and the possibility of urinary retention or ureteric stenosis. Device developers and clinicians should engage payers early to align on coverage policies that account for upfront costs but consider downstream savings from fewer surgeries and anesthesia. Adoption will likely vary between high-volume academic centers and smaller community clinics; implementation science frameworks can guide adaptation of workflows to local resources. Integrating molecular biomarkers like methylation assays or histone-modifier mutations into surveillance programs will require coordination with laboratory services and clear patient education about the implications of test results. Successful clinical integration will require coordinated partnerships across academic centers, industry sponsors, and healthcare delivery systems. Multistakeholder collaboration can accelerate evidence generation through investigator-initiated trials, optimize manufacturing and supply chains, generate real-world evidence, and develop standardized implementation pathways that facilitate broader adoption. Supply-chain resilience for polymer precursors and cross-jurisdictional regulatory compliance will be critical as manufacturing scales up.

Future recommendations

Clinical Trials

Randomized noninferiority trials comparing hydrogel-based chemoablation against TURBT in select NMIBC cohorts are needed. Specifically, a multicenter phase 3 trial stratified by European Association of Urology risk groups, with coprimary endpoints of two-year recurrence-free survival and bladder preservation rate, would provide definitive evidence. Biomarker-enriched designs (e.g., enrolling KDM6A-mutant or high methylation-signature tumors) could identify patient subsets most likely to benefit. Cooperative group networks such as the Southwest Oncology Group or the National Cancer Institute Bladder Cancer Task Force could provide infrastructure and accrual efficiency for such studies.

Bioactive Scaffolds

The development of scaffolds combining dECM with electroactive or microgrooved features to guide smooth-muscle alignment and urothelial regeneration should advance to large-animal and early human studies. First-in-human feasibility studies following GLP-compliant large-animal (porcine) models would establish safety, with collaborations between academic tissue engineering centers and regenerative medicine companies accelerating translation. Industry-academic consortia modeled after the Tissue Engineering and Regenerative Medicine International Society could facilitate standardization of manufacturing protocols and preclinical endpoints.

Local Epigenetic Therapy

Dose-finding studies of intravesical DNMT or EZH2 inhibitors delivered via hydrogels should prioritize safety and evaluate combinations with BCG or checkpoint blockade. Exploring miRNA- or CRISPR-based epigenome editors that are delivered locally is warranted. Preclinical models should include both nonmalignant and tumor-bearing bladders to assess effects on normal urothelium; off-target assessments and the reversibility of epigenetic changes are key to moving these agents toward the clinic.

Biomarker Integration

Future studies should integrate urine methylation assays and histone-modifier signatures (e.g., KDM6A mutation status) into the trial design for patient selection, risk stratification, and response monitoring. Implementation will require demonstrations of analytical validity, cost-effectiveness, and patient acceptability; researchers should evaluate whether molecular stratification improves trial efficiency by enriching for likely responders.

Manufacturing Innovation

Advances in bioprinting and microfluidics could enable custom-fitted scaffolds or drug-loaded depots tailored to individual bladder anatomy. Public-private partnerships, such as those funded through National Institutes of Health Small Business Innovation Research mechanisms or the FDA's Emerging Technology Program, could bridge the gap between prototype development and clinical-grade manufacturing while establishing regulatory pathways for personalized biomaterial devices.

## Conclusions

Hydrogel technologies are reshaping the management of NMIBC by solving the dwell-time problem and enabling sustained local delivery of chemotherapy, immunotherapies, and emerging epigenetic agents. Reverse-thermal and mucoadhesive hydrogels prolong drug retention and have achieved high complete response rates in Phase 3 trials with manageable toxicity. Epigenetic insights, such as KDM6A loss driving an ATF3-dependent program, highlight new therapeutic targets and diagnostic markers. Tissue-engineering scaffolds like GelMA and dECM provide structural support and bioactive cues for bladder regeneration. While these technologies show promise, key challenges remain: manufacturing scalability and cost for clinical-grade hydrogels, limited clinical safety data for chitosan and epigenetic modulators delivered intravesically, and the need for long-term outcomes data on scaffold-based regeneration. Near-term priorities include biomarker-stratified clinical trials, first-in-human studies of bioactive scaffolds, and health economic analyses to inform coverage policies. Integrating these advances could reduce recurrences, limit the need for repeated surgeries, and enhance the quality of life for patients with NMIBC.
